# Learning from losers

**DOI:** 10.7554/eLife.32703

**Published:** 2017-11-17

**Authors:** Benjamin Kirkup

**Affiliations:** 1 DoD CWMD Fusion CenterUS ArmyFt. BelvoirUnited States

**Keywords:** *Acinetobacter baylyi*, horizontal gene transfer, antibiotic resistance, natural competence, multi-drug resistance, microbial population dynamics, *E. coli*, Other

## Abstract

Bacteria can overcome environmental challenges by killing nearby bacteria and incorporating their DNA.

**Related research article** Cooper RM, Tsimring L, Hasty J. 2017. Inter-species population dynamics enhance microbial horizontal gene transfer and spread of antibiotic resistance. *eLife* **6**:e25950. doi: 10.7554/eLife.25950

The ruthless nature of Darwinian selection does not allow much room for carelessness. So, when a competitor eventually suffers a setback, the obvious response is to leave him in the dust and to snap relentlessly at the heels of anyone further ahead. Now, in eLife, Robert Cooper, Lev Tsimring and Jeff Hasty from the University of California, San Diego report how this may not always be the case (Cooper et al., 2017).

Some organisms, in particular bacteria, have the ability to transfer genetic material from nearby organisms, rather than just between parent and offspring. For many years it was thought that this process, also known as horizontal gene transfer, happened relatively rarely and mostly by accident. Moreover, it was thought that the recipients used the DNA mostly as a source of energy, though incorporation of foreign DNA could drive long-lasting evolutionary trends (Kurland et al., 2003; Nielsen et al., 2014; MacFadyen et al., 2001). In contrast, horizontal gene transfer is increasingly acknowledged as a routine way organisms acquire beneficial and adaptive genes, such as genes that confer resistance against antibiotics and antiseptics. One bacterium can, in effect, ‘learn’ important traits from other bacteria.

Some bacteria can speed up the process of horizontal gene transfer by killing their neighbors in order to get to their DNA. For example, the bacterium *Streptococcus pneumoniae* secretes toxins to kill sister cells or closely related bacteria, and then extracts their DNA (Steinmoen et al., 2002; Croucher et al., 2016; Wholey et al., 2016). Other bacteria, including the bacterium that causes cholera, use a contact-dependent killing mechanism known as the ‘type VI secretion system’ that involves delivering toxins directly into their victim (Borgeaud et al., 2015).

Cooper et al. placed two distantly related types of bacteria, *Acinetobacter baylyi* and *Escherichia coli*, on a surface containing nutrients over which they could grow. *E. coli* had been modified to produce a fluorescent protein in order to track its fate/whereabouts. The researchers observed that *A. baylyi* killed *E. coli* and extracted their DNA, which caused *A. baylyi* to become fluorescent. Moreover, mutant *A. baylyi* that had lost the ability to kill were still able to integrate the DNA released when their wild-type siblings killed *E. coli*. Cooper et al. showed that the extraction of DNA from *E. coli* and its integration into *A. baylyi* happened so often it could be observed in real time. *A. baylyi* that killed the *E. coli* and released its DNA increased the rate of gene transfer by several orders of magnitude compared to *A. baylyi* that had been stripped off the ability to kill. This raises the question: what can a bacterium learn from another bacterium that it has just vanquished?

Certain *Acinetobacter* bacteria thrive in hospital settings, where they can cause serious infections in compromised patients (Oberauner et al., 2013). These bacteria are also able to rapidly develop clinically-impactful antibiotic resistance (Durante-Mangoni et al., 2015). Cooper et al. showed that by killing nearby bacteria, *A. baylyi* could also acquire genes that made them resistant to antibiotics within hours. The researchers then tested two factors that could theoretically block horizontal gene transfer: enzymes that degrade DNA and irrelevant DNA. The enzymes reduced transfer rates, but the irrelevant DNA did not. Finally, a mathematical model was used to evaluate the experimental data and to simulate a range of conditions that could affect the gene transfer. The simulations showed that killing-enhanced gene transfer was more effective when *A. baylyi* outnumbered *E. coli*.

The work of Cooper et al. suggests that horizontal gene transfer rates, and as a particular case, the rate of antibiotic resistance aquisition, could be many orders of magnitude higher than previously thought, therefore challenging current antibiotic-resistance prevention strategies. Moreover, incorporating new genes and thus new abilities into a functioning genome is risky – even more so when the source genome has failed to survive. This risk is, however, minimized if the first bacterium ‘knows’ why the second one has died (that is, it has been killed by the first bacterium) and can, therefore, dismiss the cause of death as irrelevant.

Even under stressful conditions, such as exposure to antibiotics, *A. baylyi* will still encounter organisms that are able to grow. It will kill them, sample their genomes and may even locate those parts of the genome that enabled the other bacteria to survive (Sandegren and Andersson, 2009). However, it remains unclear to what extent *A. baylyi* samples the genome of the cells it has killed, and how it regulates the learning process to make it relatively ‘safe’ for themselves.

Bacteria face many selective pressures at any given moment, and most insights obtained through studying interactions between two species will not apply when several species are present (Østman and Adami, 2014; Guimarães et al., 2017). To understand why and how the bacteria learn as they do, we will need to explore DNA exchange under more realistic and multi-dimensional conditions. However, for the moment, Cooper et al. have furthered our understanding in exciting ways.
